# Effects of Trace Metal Profiles Characteristic for Autism on Synapses in Cultured Neurons

**DOI:** 10.1155/2015/985083

**Published:** 2015-02-23

**Authors:** Simone Hagmeyer, Katharina Mangus, Tobias M. Boeckers, Andreas M. Grabrucker

**Affiliations:** ^1^WG Molecular Analysis of Synaptopathies, Neurology Department, Neurocenter of Ulm University, 89081 Ulm, Germany; ^2^Institute for Anatomy and Cell Biology, Ulm University, 89081 Ulm, Germany

## Abstract

Various recent studies revealed that biometal dyshomeostasis plays a crucial role in the pathogenesis of neurological disorders such as autism spectrum disorders (ASD). Substantial evidence indicates that disrupted neuronal homeostasis of different metal ions such as Fe, Cu, Pb, Hg, Se, and Zn may mediate synaptic dysfunction and impair synapse formation and maturation. Here, we performed *in vitro* studies investigating the consequences of an imbalance of transition metals on glutamatergic synapses of hippocampal neurons. We analyzed whether an imbalance of any one metal ion alters cell health and synapse numbers. Moreover, we evaluated whether a biometal profile characteristic for ASD patients influences synapse formation, maturation, and composition regarding NMDA receptor subunits and Shank proteins. Our results show that an ASD like biometal profile leads to a reduction of NMDAR (NR/Grin/GluN) subunit 1 and 2a, as well as Shank gene expression along with a reduction of synapse density. Additionally, synaptic protein levels of GluN2a and Shanks are reduced. Although Zn supplementation is able to rescue the aforementioned alterations, Zn deficiency is not solely responsible as causative factor. Thus, we conclude that balancing Zn levels in ASD might be a prime target to normalize synaptic alterations caused by biometal dyshomeostasis.

## 1. Introduction

Autism spectrum disorders (ASD) are a group of neurological disorders currently considered to manifest from a synaptic dysfunction or synaptopathy [1]. In particular synapse formation and/or synaptic signal transduction and plasticity might be affected based on the identified candidate genes from large-scale genetic studies. However, most likely, environmental factors contribute to the etiology of ASD [2].

A strong association between imbalance in trace metal homeostasis and ASD has been reported in numerous studies [3]. Recent findings indicate that metallomic profiles of ASD patients show numerous alterations. For example, deficiencies for Zn, Ca, Fe, Mg, Mn, and Se as well as increased concentrations for Al, As, Cd, Hg, and Pb were noted in hair samples of autistic patients [4, 5] and the burden of toxic metals in patients showed a correlation with the severity of the autism phenotype [6]. This complex scenario prompted us to investigate the interplay and effects of a dyshomeostasis of different metal ions and the resultant pathological alterations of synapses.

In the past, many studies have been carried out to investigate the essentiality and toxicity of trace metals, using cells in culture [7–12]. This research has identified various trace metals considered nowadays as essential (biometals), neutral, or toxic for vertebrates. Of course, also biometals can be harmful in excessive concentrations. Thus, the difference between toxic and essential elements is based on the narrow window of concentrations, where the physiological function of biometals is seen [13]. However, trace metals do not act as separate entities influencing mechanisms or pathomechanisms in cells but exist in a careful orchestrated equilibrium [3]. To study this equilibrium that not only involves biometals but also toxic metal ions an organism is exposed to, on cellular level in vitro, we have carried out experiments on metal ions such as aluminum (Al), cadmium (Cd), copper (Cu), iron (Fe), mercury (Hg), magnesium (Mg), lead (Pb), selenium (Se), and zinc (Zn), using primary hippocampal neurons.

To test for downstream effects of imbalance of any one metal ion, which might lead to a domino effect and produces changes in all other metal ions, the present report describes the effects of heavy metal ions and the interaction among Cd, Cu, Hg, Pb, Se, and Al with Zn in cultured cells. First, we analyzed the effect of metal overload of a single trace metal on various synaptic parameters by chronic treatment of primary rat neuronal cultures with metal chlorides. Next, we determined the effect of metal overload of Cd, Cu, Hg, and Pb in combination since these metals were frequently described elevated in ASD patients. We further analyzed whether the absence of Zn and Fe, a common feature in ASD patients, modifies the effect of high Cd, Cu, Hg, and Pb levels. Finally, we investigated whether Zn supplementation is able to overcome synaptic defects induced by the trace metal profile characteristic for ASD patients.

## 2. Material and Methods

### 2.1. Materials

ZnCl_2_, CuCl_2_, CdCl_2_, FeCl_2_, SeCl_4_, AlCl_3_, MgCl_2_, HgCl_2_, and PbCl_2_ were purchased from Sigma-Aldrich. Zinpyr-1 was purchased from Sigma-Aldrich. Primary antibodies were purchased from Sigma-Aldrich (Map2, GluN1, and Shank1 for WB), Synaptic Systems (Bassoon, Homer1b/c, Shank3), Merck Millipore (GluN2a and GluN2b), and Novus Biological (Shank1 for IF). Shank2 antibodies have been described previously [14]. Secondary antibodies Alexa were purchased from Life Technologies. Unless otherwise indicated, all other chemicals were obtained from Sigma-Aldrich.

### 2.2. Hippocampal Culture from Rat Brain

The preparation of hippocampal cultures was performed essentially as described before [15] from rat (embryonic day 18; E18). After preparation the hippocampal neurons were seeded on poly-l-lysine (0.1 mg/mL; Sigma-Aldrich) glass coverslips in a 24-well plate at a density of 3 × 10^4^ cells/well or 10 cm petri dish at a density of 2.5–3 × 10^6^ cells/dish. Cells were grown in Neurobasal (NB) medium (Life Technologies), complemented with B27 supplement (Life Technologies), 0.5 mM L-Glutamine (Life Technologies), and 100 U/mL penicillin/streptomycin (Life Technologies) and maintained at 37°C in 5% CO_2_. Neurobasal medium with each of the three complements is referred to as NB+++. All animal experiments were performed in compliance with the guidelines for the welfare of experimental animals issued by the Federal Government of Germany and by the local ethics committee (Ulm University), ID number: 0.103.

### 2.3. Immunocytochemistry

For immunofluorescence, the primary cultures were fixed with 4% paraformaldehyde (PFA)/1.5% sucrose/PBS at 4°C for 20 min and processed for immunohistochemistry. After washing 2x 5 min with 1x PBS with 0.2% Triton X-100 at RT, blocking was performed with 10% FBS/1x PBS for 1 h at RT, followed by the primary antibody at RT for 2 h. After a 3x 5 min washing-step with 1x PBS, incubation with the second antibody coupled with Alexa488, Alexa568, or Alexa647 followed for 1 h at RT. The cells were washed again in 1x PBS for 10 min and counterstained with DAPI and washed for 5 min with aqua bidest and mounted with Vecta Mount.

### 2.4. Treatment of Hippocampal Cells

Cells were treated for 4 days with ZnCl_2_ (10–200 *μ*M), CuCl_2_ (10–200 *μ*M), CdCl_2_ (1–10 *μ*M), AlCl_3_ (10–500 *μ*M), HgCl_2_ (1–10 *μ*M), MgCl_2_ (10–200 *μ*M), PbCl_2_ (10–500 *μ*M), SeCl_4_ (1–10 *μ*M) and FeCl_2_ (10–400 *μ*M), or ZnCl_2_ (50 *μ*M) and CuCl_2_ (120 *μ*M) combinations, ZnCl_2_ (50 *μ*M) and CdCl_2_ (5 *μ*M) combinations, ZnCl_2_ (50 *μ*M) and HgCl_2_ (5 *μ*M) combinations, ZnCl_2_ (50 *μ*M) and PbCl_2_ (5 *μ*M) combinations, or ZnCl_2_ (50 *μ*M) and SeCl_4_ (6 *μ*M) combinations.

Metal deficient Neurobasal medium (Life Technologies) was generated using Chelex 100 Resin, 200–400 dry mesh size, sodium form (BioRad). Chelex 100 Resin was used according to the manufacturer's instructions (batch method) and the pH of the medium readjusted using hydrochloric acid. Additionally, in some conditions, original metal concentrations of some or all chelated metals were reestablished according to the Neurobasal Medium (1x) liquid media formulation using CaCl_2_, Fe(NO_3_)_3_∗9H_2_O, MgCl_2_, and ZnSO_4_∗7H_2_O. The efficacy of chelation of divalent metals was controlled by inductively coupled plasma mass spectrometry (ICP-MS) (Table 1).

### 2.5. Measurement of Trace Metal Concentrations

The trace metal concentration of growth media was measured by ICP-MS at the “Spurenanalytisches Laboratorium Dr. Baumann” (Maxhütte-Haidhof, Germany).

For fluorescent Zn-staining of cultured neurons, growth medium was discarded and the cells were washed three times with PBS. Coverslips were incubated with a solution of 5 *μ*M Zinpyr1 in PBS for 1 h at RT.

### 2.6. Protein Biochemistry

#### 2.6.1. Protein Fractionation

To obtain P2 fractions from hippocampal cultures, DIV 14 cells exposed to different compounds of interest for the indicated times were harvested and homogenized in homogenization buffer (320 mM sucrose, 10 mM HEPES, pH 7.4) containing protease inhibitor mixture (Roche). Cell debris and nuclei were removed by centrifugation at 3,200 rpm for 15 min resulting in supernatant S1 (soluble fraction) and pellet P1 (membrane associated fraction). Supernatants (S1) were centrifuged for 20 min at 11,200 rpm, resulting in S2 (soluble fraction) and P2 (crude synaptosomal fraction). The resulting pellet P2 was resuspended in homogenization buffer to perform Bradford and analyzed by western blotting.

#### 2.6.2. Western Blotting

Proteins were separated by SDS-PAGE and blotted onto nitrocellulose membranes. Immunoreactivity was visualized using HRP-conjugated secondary antibodies and the SuperSignal detection system (Pierce, Upland, USA).

### 2.7. qRT PCR

First strand synthesis and quantitative real-time-PCR amplification were performed in a one-step, single-tube format using the QuantiFastTM SYBR_Green RT-PCR kit from Qiagen according to the manufacturer's protocol in a total volume of 20 *μ*L and gene specific QuantiTect Primer Assays (Qiagen). Thermal cycling and fluorescent detection were performed using the Rotor-Gene Q real-time PCR machine (model 2-Plex HRM) (Qiagen). The SYBR Green I reporter dye signal was measured. Resulting data were analyzed using the HMBS gene as an internal standard to normalize transcript levels. All quantitative real-time PCR reactions were run in technical triplicates.

### 2.8. Statistic

#### 2.8.1. Synapse Measurement

Acquisition and evaluation of all images were performed under “blinded” conditions. For cell culture experiments 10 cells of each condition were imaged. Fluorescence images were obtained using an upright Axioscope microscope equipped with a Zeiss CCD camera (16 bits; 1280 × 1024 ppi) using Axiovision software (Zeiss) and ImageJ 1.49i. Statistical analysis was performed using Microsoft Excel for Macintosh and tested for significance using *t* tests (all values were normally distributed).

#### 2.8.2. Western Blot Quantification

Evaluation of bands from Western blots (WBs) was performed using ImageJ. Three independent experiments were performed and blots imaged using a MicroChemi Imaging System from Biostep. The individual bands were selected and the integrated density was measured. All WB bands were normalized to *β*-III-tubulin or beta-actin and the ratios averaged and tested for significance with a level of significance set at 0.05 (<0.05^*^; <0.01^**^; <0.001^***^).

#### 2.8.3. qRT PCR Quantification

Relative quantification is based on internal reference genes to determine virtual mRNA levels of target genes. Cycle threshold (Ct) values were calculated by the Rotor-Gene Q Software (version 2.0.2). Ct values were transformed into virtual mRNA levels according to the formula: virtual mRNA level = 10 ∗ ((Ct_(target)_ − Ct_(standart)_)/slope of standard curve).

## 3. Results

**Figure 1:**
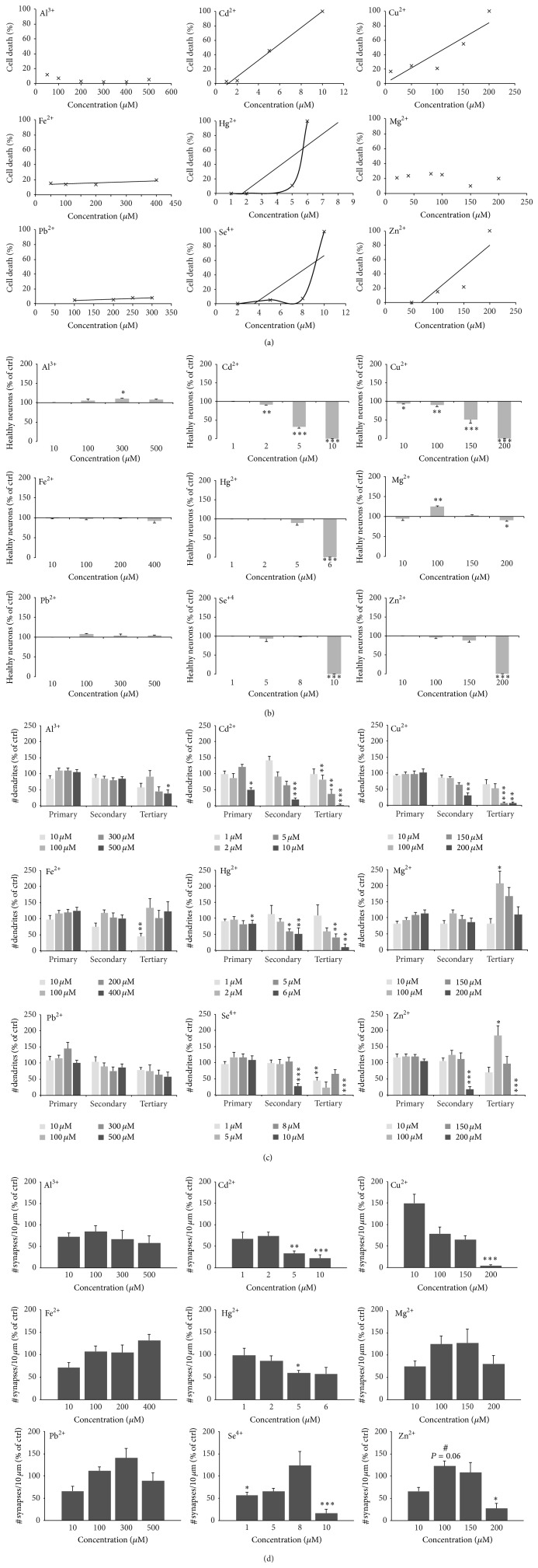
Toxicity profiles of trace elements in primary hippocampal neuronal cell culture. ((a), (b)) The amount of cell death was calculated using various concentrations for each trace metal by assessing the number of neuronal apoptotic nuclei (identified by MAP2 and DAPI staining) per optic field (from 5 fields of view and *n* = 3 cultures) normalized against the total number of neurons per optic field. The results show no changes in cell health over a wide spectrum of concentrations for Al, Fe, and Pb, and a linear correlation between metal level and cell death for Cd, Cu, and Zn. A linear correlation might also exist for Hg and Se although an exponential increase in cell death beyond a certain concentration is more likely. Addition of Mg increased cell survival in a low concentration range and with higher concentrations leading to cell death (a). (b) Using *t*-tests, *P* values were calculated to evaluate, whether changes between different concentrations compared to untreated control cells are significant. (c) As an alternative read-out, the number of primary, secondary, and tertiary dendrites was investigated. Cells were stained with MAP2 antibody. As signs of cell death, neurons show fragmentation (pinching off) dendrites, starting with branches more distal from the soma. Dendrites showing signs of fragmentation were not counted. High concentrations of metals that were shown to lead to cell death ((a), (b)) lead to a reduction in dendritic branching starting from tertiary dendrites and also affecting secondary and primary in the most toxic cases. Although, for some metal, no significant cell death in a certain concentration was seen, and already a fragmentation of dendrites is visible (e.g., 5 *μ*M Hg). Mg and Zn show a significant increase in dendritic branching restricted to tertiary dendrites in a lower concentration range. (d) To assess whether some trace elements display a synaptogenic effect, synapses were labeled using Bassoon fluorescence and the number of Bassoon positive puncta was measured per 10 *μ*m dendrite length on primary dendrites (3 dendrites per cell, 10 cells in total per metal and concentration). While synapse number is decreased correlating with toxic effects for most metal ions, such as Cd, Cu, and Se, an increase in synapse number can be seen for Fe and lower concentrations of Mg and Zn (seen as a trend). ((a)–(d)) All cells were treated with metals between DIV 10 and DIV 14 before analysis.

In our previous study [3], we reported characteristic biometal profiles in neurological disorders. In ASD, several trace metals are known to be either depleted or to occur in excess (Table 2). Therefore, to understand the consequences of all these alterations in trace metals for synapses, in a first set of experiments, we aimed to analyze first the influence of each trace metal on cell health and synapse numbers separately (Figures S1A and B in Supplementary Material available online at http://dx.doi.org/10.1155/2015/985083 and Figure 1). To that end, we treated hippocampal neuronal cell cultures between DIV 10 and DIV 14 with different concentrations of the trace metals Al, Cd, Cu, Fe, Hg, Mg, Pb, Se, and Zn. As expected, a linear correlation between metal levels and the amount of cell death was found, especially for Cd, Cu, and Zn (Figures 1(a) and 1(b)). However, not all metals display this correlation. We could not detect significant cell death over a wide spectrum of concentrations for Al, Fe, and Pb. Additionally, for Hg and Se, an exponential increase in cell death beyond a certain concentration was observed. Exposure of cells to Mg increased cell survival in a low concentration range and with higher concentrations leading to cell death (Figures 1(a) and 1(b)). Treatment with mannitol was used to exclude the influence of osmotic stress on cell health (Figure S1C). Based on the correlation between cell health and the concentration of a certain trace metal applied, LD_50_ concentrations were calculated for each metal (Figure S1D). Glial cells that were present to a low extent in the neuronal cultures were much more resistant to alterations in trace metal concentrations and showed no signs of cell death in the toxic concentration range seen for neuronal cells (Figure S1E).

Furthermore, the number of primary, secondary, and tertiary dendrites of neurons after treatment was investigated. Given that neurons show a fragmentation of dendrites, starting with branches more distal from the soma as first sign of affected cell health, a decrease in dendritic branching might reveal cells that did not show apoptotic nuclei yet already suffered from the exposure to trace metals. Similar to the results shown above, high concentrations of metals that were shown to lead to cell death led to a reduction in dendritic branching starting from tertiary dendrites and also affecting secondary and primary in the most toxic cases (Figure 1(c)). Indeed, although, for some metal, no significant cell death in a certain concentrations was seen, already a fragmentation of dendrites is visible. Interestingly, supplementation of Mg and Zn in a low concentration leads to a significant increase in dendritic branching restricted to tertiary dendrites (Figure 1(c)).

The loss of synapses is an accompanying factor of neurodegeneration. Therefore, a decrease in synapse number is expected to correlate with the toxic effects of trace metals and the resulting cell death. Indeed, the quantification of synapse density in treated neurons and compared to untreated control cells shows a correlation of toxic effects and synapse loss for metal ions, such as Cd, Cu, and Se (Figure 1(d), Figure S1F). However, an increase in synapse number can be seen for Fe and lower concentrations of Mg and Zn (Figure 1(d), Figure S1F).

**Figure 2:**
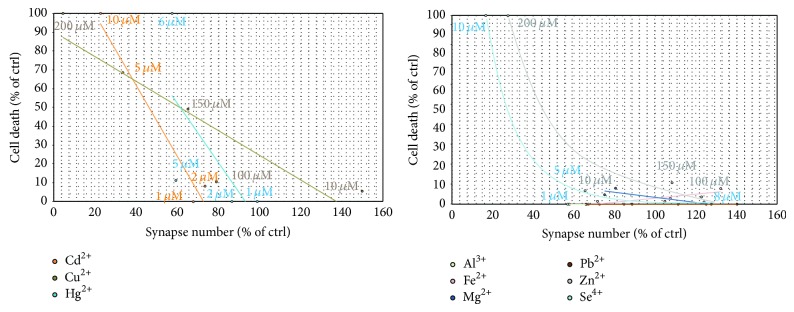
Effect of trace metals on synapse formation or stability. While the observed decrease in synapse number can be correlated with the overall cell death of neurons in culture for Cd, Cu, and Hg (left panel), no correlation was found for Al, Fe, and Pb that did not significantly alter cell health. Mg shows only a weak correlation due to its nontoxic effects on neurons over a wide concentration range. Both Zn and Se may display even increased synapse numbers despite ongoing cell death.

Since, for some metal ions, we could determine an effect on synapses independent of a possible cellular toxicity (Figure 2), a role of these metals in synapse formation and/or stabilization is likely. For example, Mg shows only a weak correlation due to its nontoxic effects on neurons over a wide concentration range. Additionally, supplementation of both Zn and Se, in low concentrations increased synapse density, which then can be found increased compared to untreated control despite ongoing cell death.

Based on the findings described above, it might be possible that the absence of a synaptogenic effect of some metals such as Zn and the toxic effects of others act in combination in ASD when deficiencies and overload of certain trace metals occur in parallel. Therefore, we established a biometal profile resembling the assumed trace metal alterations in ASD patients and evaluated the consequences on cell health and especially on synapses.

Hippocampal neurons were grown from DIV 10 to DIV 14 in cell culture media containing different sets of trace metals: control cells (Ctrl) were grown in trace metal depleted Neurobasal medium that was reconstituted for all depleted trace metals (Mg, Ca, Fe, and Zn) according to the manufacturer's indicated metal concentrations. In none of the readouts, we could observe differences between original and reconstituted Neurobasal medium (Figure S2). Additionally, cells were grown in Neurobasal medium with addition of putative toxic metals (0.5 *μ*M Cd, Cu, Hg, and 2 *μ*M Pb) found to be increased in ASD patients (ASD1). Control cells for this condition were grown in Neurobasal medium. In a third condition, cells were grown in trace metal depleted Neurobasal medium that was reconstituted only for Mg and Ca, with addition of the putative toxic metals (0.5 *μ*M Cd, Cu, Hg, and 2 *μ*M Pb) (ASD2). ASD2 growth medium, therefore, with Fe and Zn deficiency and overload of Cd, Cu, Hg, and Pb resembles the characteristic biometal profile found in ASD [3].

**Figure 3:**
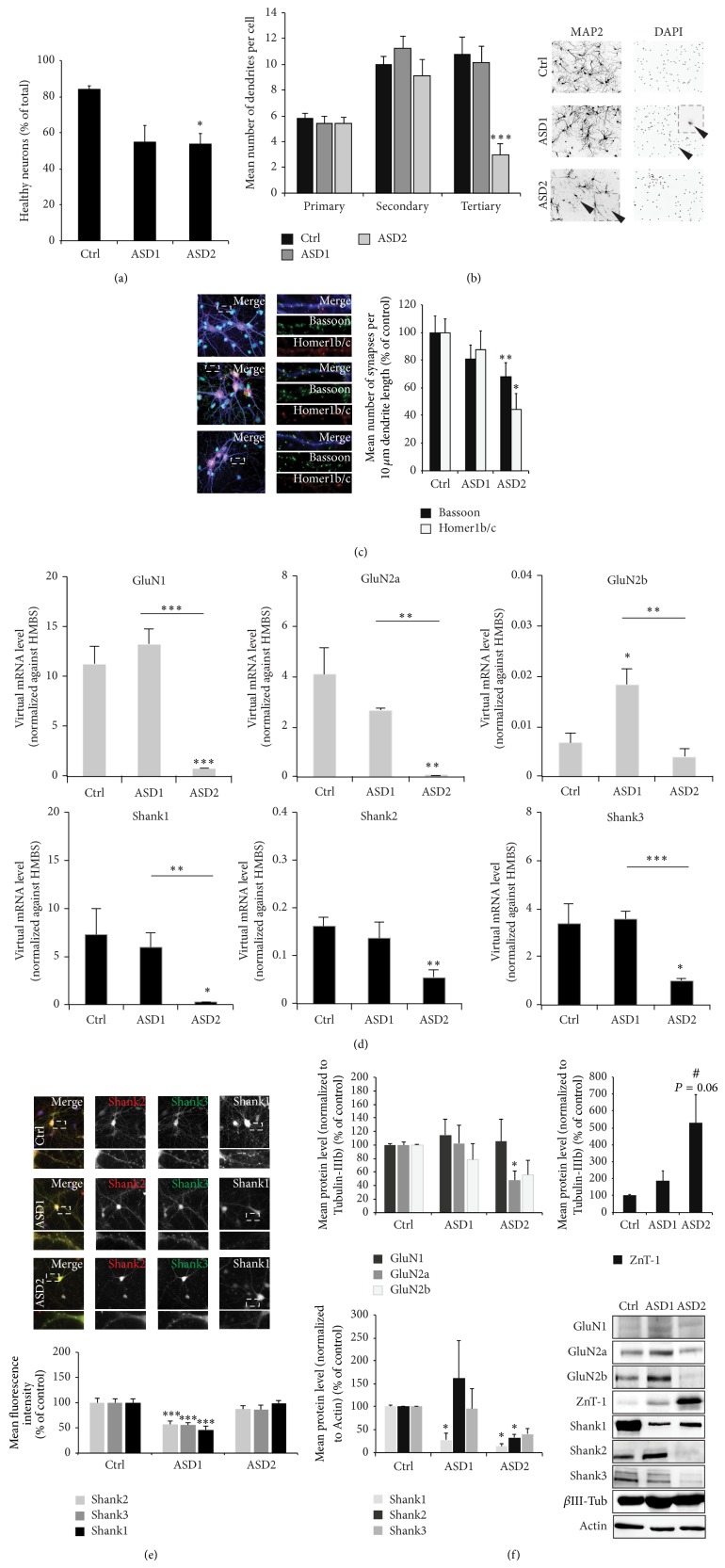
An ASD biometal profile affects cell health and synapse numbers* in vitro*. (a) Hippocampal neurons were grown from DIV 10 to DIV 14 in cell culture media containing different sets of trace metals: control cells (Ctrl) were grown in Neurobasal medium. ASD1 cells were grown in Neurobasal medium with addition of putative toxic metals (0.5 *μ*M Cd, Cu, Hg, and 2 *μ*M Pb). ASD2 cells were grown in trace metal depleted Neurobasal medium that was reconstituted only for Mg and Ca, with addition of the putative toxic metals (0.5 *μ*M Cd, Cu, Hg, and 2 *μ*M Pb). The amount of cell death was calculated assessing the number of neuronal apoptotic nuclei (identified by MAP2 and DAPI staining) per optic field (from 5 fields of view) normalized against the total number of neurons per optic field. A significant reduction in cell health can be observed in cells deficient in Fe and Zn and subjected to toxic metals (ASD2). (b) The number of primary, secondary, and tertiary dendrites was investigated from 10 cells per condition. Cells were stained with MAP2 antibody. As signs of cell death, neurons show fragmentation (pinching off) dendrites, starting with branches more distal from the soma. Dendrites showing signs of fragmentation were not counted. Corresponding to the increase in cell death, neurons growing under ASD2 conditions showed significantly increased signs of dendritic fragmentation. (c) Synapses were labeled using Bassoon and Homer1b/c fluorescence and the number of immunoreactive puncta was measured per 10 *μ*m dendrite length on primary dendrites (3 dendrites per cell, 10 cells in total per group). Merged images show additional staining of nuclei using DAPI (cyan) and MAP2 (blue). A significant reduction can be seen in cells growing in medium resembling the biometal profile found in ASD patients (ASD2). (d) Expression levels of NMDA receptor subunits (GluN1, GluN2a, and GluN2b) and SHANK genes (Shank1, Shank2, and Shank3) were measured by qRT-PCR. Virtual mRNA concentrations are shown averaging from three replicates and normalized against HMBS. A significant decrease of GluN1 and GluN2a mRNA expression levels can be seen in cells grown under ASD2 conditions. Under ASD1 conditions, GluN2b levels significantly increase. A significant reduction in gene expression levels can also be observed in Shank family members under ASD2 conditions, which is significant for Shank1, Shank2, and Shank3. (e) Immunocytochemistry of hippocampal neurons DIV 14 grown under control, ASD1, and ASD2 conditions. The fluorescence intensity of Shank positive puncta was measured using antibodies specific for Shank1, Shank2, and Shank3. Exemplary images (upper panel) and quantification of average puncta signal intensity of 10 cells per condition (lower panel). Merged images show additional DAPI staining of the nucleus. Neurons grown under ASD1 conditions show a significant decrease of synaptic Shank proteins, while neurons under ASD2 conditions did not show a reduction. (f) Analysis of protein expression levels in synaptic (P2) fractions of NMDAR subunits, proteins of the Shank family, and ZnT-1 from three independent experiments normalized against *β*-III-tubulin or actin. Neurons grown in ASD2 medium show a significant reduction of GluN2a receptor subunits and a trend towards a decrease of GluN2b. In contrast, synaptic ZnT-1 shows a strong upregulation. The expression of Shank family members is reduced under ASD2 conditions (lower left panel). The reduction is significant for Shank1 and Shank2 and seen as a clear trend for Shank3. Exemplary bands are shown in the lower right panel.

Cells exposed to ASD2 medium indeed displayed a significant reduction in cell health visible by an increased number of apoptotic nuclei and increased dendritic fragmentation (Figures 3(a) and 3(b)). The number of synapses per 10 *μ*m dendrite length assessed by quantification of Bassoon and Homer1b/c positive puncta was significantly reduced in cells growing in medium resembling the biometal profile found in ASD patients (ASD2) (Figure 3(c)). Along with a reduction in the number of synapses, gene expression levels of synaptic receptors such as NMDAR (GluN1, GluN2a, and GluN2b) as well as the Zn dependent Shank scaffold proteins (Shank1, Shank2, and Shank3) show significant alterations (Figure 3(d)). While the presence of putative toxic metals increased in ASD patients (ASD1) is sufficient to significantly increase GluN2b expression levels, GluN1 and GluN2a and Shank1, Shank2, and Shank3 mRNA levels are only significantly altered in case of an additional Zn and Fe deficiency (ASD2). Fluorescent readouts for Shank proteins show a reduction on protein level under ASD1 conditions, while the remaining synapses under ASD2 condition display normal Shank levels (Figure 3(e)). However, analysis using protein biochemistry shows that, overall, Shank protein concentrations are reduced in the P2 fraction of cells growing under ASD2 condition (Figure 3(f)). Additionally, similar to the decrease of mRNA levels of NMDAR subunits, we detected significantly less GluN2a protein and a trend towards a decrease of GluN2b protein levels in cells grown in ASD2 medium. Furthermore, ZnT-1 expression levels, a Zn exporter recently described as being enriched at postsynapses [16], reacts very sensitively to changes in trace metal concentrations as those induced by application of ASD2 medium (Figure 3(f)).

**Figure 4:**
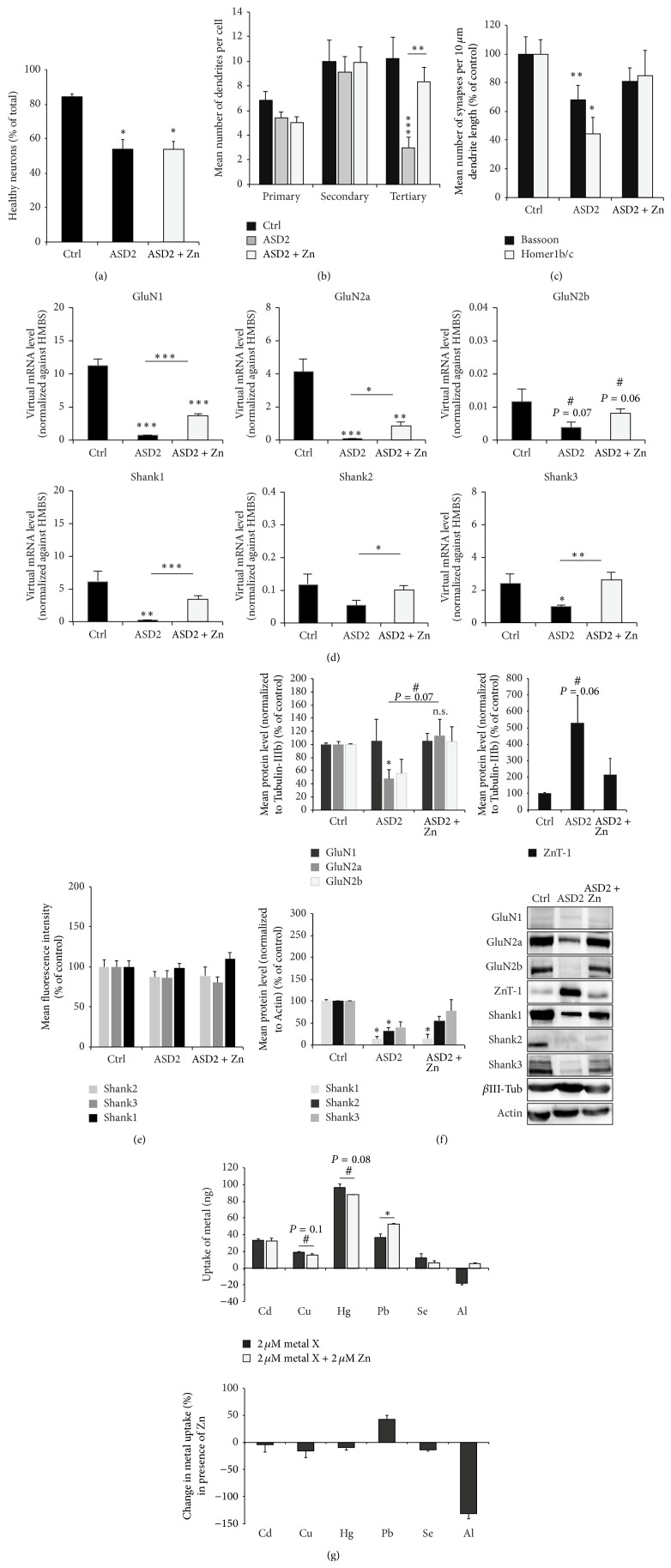
Synergistic and antagonistic relationships between trace metals and Zn. Hippocampal neurons were grown from DIV 10 to DIV 14 in cell culture media containing different sets of trace metals: control cells (Ctrl) were grown in trace metal depleted Neurobasal medium that was reconstituted for all depleted trace metals (Mg, Ca, Fe, and Zn) according to the manufacturer's indicated metal concentrations. ASD2 cells were grown in trace metal depleted Neurobasal medium that was reconstituted only for Mg and Ca, with addition of the putative toxic metals (0.5 *μ*M Cd, Cu, Hg, and 2 *μ*M Pb). ASD2 + Zn cells were grown in ASD2 medium except that 50 *μ*M Zn was added to the medium. The amount of cell death was calculated assessing the number of neuronal apoptotic nuclei (identified by MAP2 and DAPI staining) per optic field (from 5 fields of view) normalized against the total number of neurons per optic field. A significant reduction in cell health can be observed in cells deficient in Fe and Zn and subjected to toxic metals (ASD2). Zn addition was unable to rescue the observed cell death (ASD2 + Zn). (b) The number of primary, secondary, and tertiary dendrites was investigated from 10 cells per condition. Cells were stained with MAP2 antibody. As signs of cell death, neurons show fragmentation (pinching off) dendrites, starting with branches more distal from the soma. Dendrites showing signs of fragmentation were not counted. The significant reduction seen under ASD2 conditions could be rescued significantly by addition of Zn. (c) Synapses were labeled using Bassoon and Homer1b/c fluorescence and the number of immunoreactive puncta was measured per 10 *μ*m dendrite length on primary dendrites (3 dendrites per cell, 10 cells in total per group). A significant reduction can be seen in cells growing in medium resembling the biometal profile found in ASD patients (ASD2). Addition of Zn rescues the effects seen on synapse density. (d) Expression levels of NMDA receptor subunits (GluN1, GluN2a, and GluN2b) and SHANK genes (Shank1, Shank2, and Shank3) were measured by qRT-PCR. Virtual mRNA concentrations are shown averaging from three replicates and normalized against HMBS. Supplementation of Zn was able to significantly increase the reduction in mRNA levels seen in cells growing under ASD2 conditions. However, in all cases, mRNA levels still remained significantly decreased compared to controls for NMDAR subunits GluN1 and GluN2a. The expression levels of Shank family members were rescued completely (Shank1 and Shank3). (e) Zn supplementation on DIV 10–14 did not significantly alter fluorescence intensities of synaptic puncta. (f) Analysis of protein expression levels in synaptic (P2) fractions of NMDAR subunits, proteins of the Shank family, and ZnT-1 from three independent experiments normalized against *β*-III-tubulin or actin. Neurons grown in ASD2 medium show a significant reduction of GluN2a receptor subunits and a trend towards a decrease of GluN2b, which is rescued by supplementation of Zn. Similarly, synaptic ZnT-1 levels decrease almost to control levels in ASD2 medium with Zn compared to ASD2 Zn deficient medium. The expression of Shank proteins is reduced under ASD2 conditions and the synaptic concentration of Zn-dependent Shank2 and Shank3 family members is restored by Zn supplementation such that Shank2 levels are no longer significantly decreased under ASD2 + Zn condition (lower left panel). Exemplary bands are shown in the lower right panel. (g) Using ICP-MS, the extracellular metal concentrations of hippocampal cell cultures (*n* = 3 per metal, Mann-Whitney *U* test) was measured with and without the presence of Zn. A reduction in the uptake of Cu, Hg, and Se was seen as trend upon coapplication of Zn, while an increase in uptake of Pb was visible.

Finally, in a last set of experiments, we wanted to determine, whether the addition of a single specific trace metal might lead to a rescue in the observed phenotype after inducing an ASD like biometal profile in cell culture or whether all alterations need to be addressed separately. Given that an antagonistic role of Zn and Pb or Cu was proposed before [3, 4] and that we could verify a potentially beneficial effect of Zn on synapses, we next wanted to elucidate whether Zn supplementation in combination with harmful metals (ASD2) is able to modify the toxicity of the trace metal on neurons* in vitro*. To that end, we performed similar readouts as described above (Figure 3). However, ASD2 medium (Neurobasal medium that was reconstituted only for Mg and Ca, with addition of the putative toxic metals (0.5 *μ*M Cd, Cu, Hg, and 2 *μ*M Pb)) was supplemented with 50 *μ*M Zn. Addition of Zn was unable to rescue the observed cell death detected in cultures grown under ASD2 conditions (Figure 4(a)), although it led to a significant reduction in dendritic fragmentation (Figure 4(b)). Along with this, the reduction in the number of synapses per 10 *μ*m dendrite length assessed by quantification of Bassoon and Homer1b/c positive puncta seen under ASD2 conditions was rescued after addition of Zn (ASD2 + Zn) (Figure 4(c)). Supplementation of Zn was also able to significantly increase the reduction in mRNA levels of NMDA receptor subunits and Shank family members seen in cells growing under ASD2 conditions (Figure 4(d)). Shank mRNA expression levels were no longer significantly different from those of cells growing under control conditions. However, in case of NMDA receptor subunits, mRNA levels still remained significantly decreased compared to controls. The observed changes on protein level of NMDA receptor subunits, the Zn binding Shank2 and Shank3, and ZnT-1 in turn were completely rescued after addition of Zn (ASD2 + Zn) (Figure 4(f)). Immunofluorescence readouts revealed no changes in synaptic protein levels after supplementation of cells growing under ASD2 conditions with Zn (Figure 4(e), Figure S2D).

Furthermore, we used ICP-MS to measure the extracellular metal concentrations of hippocampal cell cultures with and without the presence of Zn. A reduction in the uptake of Cu, Hg, and Se was visible upon coapplication of Zn, while an increase in uptake of Pb was visible (Figure 4(g)). When ZnCl_2_ (50 *μ*M) was combined with either CdCl_2_ (5 *μ*M), CuCl_2_ (120 *μ*M), HgCl_2_ (5 *μ*M), PbCl_2_ (5 *μ*M), or SeCl_4_ (6 *μ*M) treatment, a slight decrease of neuronal cell death upon exposure to ZnCl_2_ and HgCl_2_ compared to the exposure to HgCl_2_ alone was observed. Further, a significant improvement of neuronal cell viability was identified if ZnCl_2_ and CdCl_2_ were supplemented together. There, zinc could restore neuronal cell health status close to control levels (Figures S2E, F).

The alterations observed cannot be explained by changes in transsynaptic Zn signaling given that presynapses in hippocampal cell culture have very low to absent levels of vesicular Zn (Figure S2G). Additionally, the alterations caused under ASD2 conditions—although to high extent rescued by Zn supplementation—are not based on Zn deficiency alone. A comparison between ASD2 and pure Zn deficient (ZnD) conditions reveals that Zn deficiency without additional alterations in biometals does not lead to the significant reduction in cell health (Figures S3A, B) and synapse number (Figure S3C) such as seen under ASD2 conditions. Furthermore, the decrease in mRNA expression levels of NMDA receptor subunits is less pronounced and absent for SHANK family members under pure Zn deficiency (Figure S3D). However, as reported before [14], Zn deficiency significantly affects the synaptic levels of the Zn binding Shank family members Shank2 and Shank3 using immunofluorescent readouts (Figure S3E). Protein biochemistry with P2 fractions (Figure S3F) reveals that, on protein level, Zn deficiency alone already affects NMDAR subunit expression, but to a lesser extent the expression of Shanks.

## 4. Discussion

An increasing number of reports of imbalanced trace metal homeostasis in children that suffer from neurodevelopmental disorders raise the need for a better understanding of how putative toxic metals affect neuronal cells. Therefore, we investigated the effects of different essential and nonessential trace metals on neurons* in vitro* and further examined their relationship with zinc, one of most abundant trace metals in the brain.

In a first set of experiments, we investigated the influence of overload with a single trace element. Although trace metals such as Mg, Cu, Fe, Se, and Zn are essential with a vital role in normal brain functions, increased levels in the brain may lead to severe neurological symptoms. However, we found a strong resistance of neurons against FeCl_2_ induced toxicity with the absence of morphological changes even at very high concentrations (up to 400 *μ*M) and a slight dose-dependent synaptogenic effect. This absence of neuronal cell death is in line with the results from Bishop et al. [17] reporting that the exposure to 100 *μ*M of ferric ammonium citrate for 24 h to cultured neurons increased the intracellular Fe content by factor 30 but did not affect cell viability [17].

Similarly, only few pathologically morphological changes of primary neurons could be seen after exposure to MgCl_2_ in the tested concentration range. Similar findings were reported by Regan et al. [18] who treated primary cortical neurons with up to 3 mM MgCl_2_ without detecting any morphological evidence of cell injury [18]. In our study, intermediate concentrations (100 *μ*M) of MgCl_2_ increased the number of tertiary dendrites and slightly increased the number of synapses. In contrast, exposure of neurons to CuCl_2_ leads to a dose-dependent cell death and a significant reduction of synapse numbers correlated to the reduced number of viable cells, which can be also observed in younger neurons at DIV 6 [19].

Se is an essential trace metal with a narrow range of concentrations between deficiency and toxicity. Also, in this study, total neuronal cell death has been observed at 10 *μ*M SeCl_4_, whereas 8 *μ*M SeCl_4_ were shown to increase synapse numbers. Little is known about the toxic effect of SeCl_4_ on cultured neuronal cells due to the fact that most studies concentrated on the protective effect of Se against glutamate induced excitotoxicity [20] or against the toxicity of inorganic mercury [21]. Savaskan et al. [20] have reported that the exposure to 10 *μ*M sodium selenite decreased HT22 cell viability about 20%. Cell death did not reach 100% until 1 mM [20]. These differences are likely due to the use of different cell culture systems and it is not surprising that an immortalized hippocampal cell line is much more resistant against Se induced toxicity than primary neurons.

Zn is involved in a huge variety of cellular processes but an increased concentration of unbound Zn ions has been reported to be especially harmful to the central nervous system and to kill cells* in vitro* [22]. In this study, concentrations above 100 *μ*M ZnCl_2_ induced neuronal cell death. However, Zn supplementation led to an increase in synapse numbers.

In contrast to essential trace metals, exposure to putative toxic metals is expected to be much more harmful. Indeed, even low concentrations of CdCl_2_ had severe effects on neuronal cell health (LD_50_ 5 *μ*M). We observed a dose-dependent neuronal cell death and a linear correlated reduction of synapses. Cd toxicity was shown to lead to impaired neurogenesis, reduced neuronal differentiation, and reduced axogenesis* in vitro* [23] and even lower concentrations have been reported to repress dendritic and synaptic development [24, 25]. Despite fatal neuronal cell death, at low Cd concentrations, glial cells seem to be more resistant which is in accordance with previous reports [26].

Although Al is a well-accepted neurotoxin [27] and discussed as factor for the development of neurodegeneration, for example, as seen in Alzheimer's disease (AD) [28], we did not observe adverse effects on neurons and synapses even at very high concentrations of AlCl_3_ (up to 500 *μ*M) similar to a study by Kawahara et al. [29]. However, lipophilic aluminum species like aluminum acetylacetonate or aluminum maltolate might be more toxic than AlCl_3_ [29]. Additionally, a possible precipitation of AlCl_3_ in physiological pH [27] might prevent Al from reaching intracellular concentrations high enough to induce toxicity. It is possible that Al does not affect the number of synapses but rather alters synaptic plasticity [30, 31] or may act in addition to other predisposing factors. Intriguingly, it was proposed that the genetic component of AD pathology might involve a susceptibility gene, yet, to be identified, that increases Al absorption [32].

Hg causes neurocognitive deficits and neuromotor disabilities [33–36]. Thus it is not unexpected that elevated Hg levels led to severe neuronal cell death already at low concentrations (LD_50_ 5 *μ*M) accompanied with a dose-dependent reduction of synaptic density. Hg toxicity has been reported to increase with incubation time [37]; thus, even lower concentrations of HgCl_2_ (25–100 nM) can lead to significantly reduced neuronal cell survival if exposure is expanded [38].

Pb has been known to be an environmental contaminant associated with several neurological diseases and has been reported to affect a child's developing brain adversely [39, 40]. However, in this study, Pb seemed to act slightly beneficially on neuronal cell health and slightly increased synaptic number at higher concentrations. Similarly, Audesirk et al. [41] have reported that Pb showed the least effects on hippocampal neurons at intermediate concentrations (1–10 *μ*M) [41]. It might be speculated that the toxicity of Pb is not caused by a direct influence on neurons but by interference with processes already outside the CNS—possibly due to the absence of certain receptors or competition with target proteins. A toxic interference, as reported, for example, for Cu and Zn [42], might happen already within the gastrointestinal system and not within the brain.

It should be mentioned that many factors such as the use of FBS or B27 in the growth medium might influence the toxicity of certain metals. For example, bovine serum albumin (BSA) present in fetal bovine serum (FBS) and B27 supplement has high Zn binding capacity [43] and the actual concentration that reaches the neuron is hard to predict. Indeed, it was shown that a concentration of more than 100 nM “free” Zn is toxic for cells [44]. Based on this and the calculated value for Zn in these experiments, it is likely that a high amount of trace metals will be buffered and bound to proteins immediately after application.

Already the manipulation of a single trace metal shows an influence on cell viability but also synapses. However, in patients such as those suffering from ASD, a combination of trace metal alterations (excess and deficiencies) occurs that might establish a whole new environment for neurons and affect synapse number and function. Thus, in a second set of experiments, we established a biometal profile that resembles the one reported in individuals with ASD and assessed the effect on cell health and synapse density and composition.

Recently, a pathway at excitatory glutamatergic synapses was identified by large-scale genetic screens and the description of ASD candidate genes [45] that is centered around proteins of the Shank family. Mutations in the three family members Shank1, Shank2 (ProSAP1), and Shank3 (ProSAP2) have been described to be associated with ASD [46, 47] and animal models with deletions of these genes displaying ASD like phenotypes [48, 49]. However, Shank2 and Shank3 are also proteins that are highly regulated by Zn-binding [50] and their amount reduced in animal models for Zn deficiency [14] or Cu overload [51]. Therefore, these proteins might be promising candidates providing a link between trace metal imbalances and synaptic defects in ASD. Based on this model, here, we evaluated synaptic Shank protein levels. Indeed, we observed a loss of synapses along with a reduction of P2 associated protein levels in Shank family members.

Given that Zn is one of the most abundant trace metals in the brain and acts at the postsynapse influencing synaptic proteins and plasticity [52–55], we investigated whether Zn might be able to rescue the ASD biometal profile induced phenotype. Chronic Zn supplementation was able to normalize the alterations in Zn-binding Shank2 and Shank3 but did not further increase Shank levels as reported to result from acute Zn supplementation [14]. Thus, feedback mechanism might be involved that regulates Shank levels over time limiting them to the level seen in controls.

Additionally, we were interested in levels of NMDAR subunits since alterations such as a decrease of NMDARs was described in Shank3 deficient cells, Shank2 knock-out mice, and prenatal Zn deficient mice that also display a reduction of Shank2 and Shank3 [14, 56, 57]. Indeed, similar to the expression of Shank proteins, expression of NMDAR subunits was sensitive to trace metal profiles such as those seen in ASD patients, in particular increased levels of putative toxic metals in combination with Zn and Fe deficiency (ASD2), on mRNA and synaptic protein level. This again argues that alterations in trace metal status as seen in ASD patients—if present in a similar manner in the brain of patients—might be sufficient to induce changes similar to those seen in genetic models of ASD such as Shank knock-out mice. In particular, a shift from NMDAR subunit 2b (GluN2b) containing synapses to GluN2a as seen in normal development might be affected by the reduction in GluN2a but not GluN2b expression on mRNA and protein level that happens only in the trace metal profile similar to that seen in ASD patients.

After Zn supplementation, mRNA levels of NMDA receptor subunits remained significantly decreased compared to controls. However, compared to ASD2 conditions, the expression levels were significantly rescued. In contrast, gene expression of Shank family members is fully restored after Zn supplementation. The mechanisms of how Zn might influence NMDAR and Shank transcription are currently not well understood. However, Myc-associated zinc finger protein (MAZ) binds a GC box element in the regulatory regions of GluN1, GluN2A, GluN2B, and GluN2C [58]. Thus, availability of Zn might affect, among many other transcription factors with zinc finger domains, MAZ, thereby affecting NMDAR subunit expression rates. Another transcription factor that is sensitive to trace metal levels, especially to Zn, is MTF1 (metal-regulatory transcription factor 1). MTF1 binds to metal response elements (MREs) within the promoter region of genes in presence of Zn to increase their transcription rate [59, 60]. Intriguingly, the promoter of Shank3 contains a MRE. However, whether gene regulation of Shanks by Zn is dependent on MTF1 has not been investigated so far.

The obtained results from gene expression analyses did not always correlate with protein expression measured by Western blot analysis and analysis of immunofluorescence intensity. However, for mRNA analyses, total RNA from whole cell lysate was taken. In contrast, Western blot analyses were performed using synaptosome enriched P2 fractions. It is thus possible that a decrease of mRNA level might be reflected by a decrease in extrasynaptic proteins. Additionally, the decrease on mRNA levels might not have been present long enough to affect the concentration of stable proteins with slow turn-over rates. Similarly, in fluorescence readouts, only synaptic immunoreactive puncta were measured. Here, almost complete loss of proteins leading to very low signal intensities below background level might not have been detected.

Furthermore, we analyzed the synaptic expression of ZnT-1 that was recently reported to be enriched at the PSD and that is associated with NMDAR [16]. We found that protein levels of ZnT-1 react sensitively to the established trace metal profiles, again especially to ASD2. One might speculate that export of Zn as synaptic acting signal, which might be decreased in Zn depleted medium, is compensated by an increased number of ZnT-1 proteins at the synapse. However, given that ZnT-1 global expression usually is upregulated in response to increasing Zn concentrations [61], a novel mechanism of synaptic ZnT-1 regulation is implied. Intriguingly, the upregulation of Zn-T1 is reduced after replenishment of ASD2 medium with Zn.

Although the alterations observed cannot be explained by Zn deficiency only, our data show that supplementation of ASD2 medium with Zn alone is already sufficient to rescue most of the effects caused by exposure to putative toxic trace metals along with Zn and Fe deficiency. Addition of Zn could partly restore cell health (reduced branching of tertiary dendrites) and synaptic loss. Additionally, to an effect on synapses, the toxicity of Pb, Cd, Cu, and Hg might be reduced upon Zn treatment due to an antagonistic relationship between Zn and these trace metals. In accordance with the literature [62, 63], we could observe a strong and significant antagonistic behavior of Zn and Cd on neuronal cell health and synapse density. Further, a slight reduction of Cu and Hg uptake has been seen upon coapplication of ZnCl_2_ that might be further increased if the Zn concentration would not be equimolar to Cu and Hg but exceed their concentration.

Taken together, our results show that, independent of the important but so far unsolved question, whether these imbalances in trace elements seen in ASD patients are cause or consequence, indeed an altered biometal status may have an influence on synapse composition and function. Addressing the dysregulation of trace metals might be a novel approach in modifying the disease phenotype in patients and should be thoroughly investigated* in vivo* in future studies.

## Supplementary Material

Figure S1: Exemplary images of toxicity profiles of trace elements in primary hippocampal neuronal cell culture, glia cell culture and calculation of LD_50_ values for each trace element. Furthermore, definition of dendritic branching analysis and evaluation of osmotic pressure effects on neuronal cell health.
Figure S2: Comparison of original and reconstituted NB+++ Medium and exemplary images of immunofluorescence intensities of Shank proteins under Ctrl, ASD2 and ASD2+Zn conditions. Further, analysis of antagonistic or synergistic relation between zinc and toxic trace elements and examination of presence of presynaptic zinc.
Figure S3: Pure zinc deficiency is not causative for effects observed under ASD2 conditions.

## Figures and Tables

**Table 1:** Depletion of divalent metal ions by Chelex 100 purification of growth media. Chelex chelating ion exchange resin has high preference for copper, iron, zinc, and other divalent heavy metals over monovalent cations such as sodium and potassium with a selectivity for divalent over monovalent ions of approximately 5,000 to 1. To control the efficacy of ion chelation, the Zn concentration of Neurobasal medium was measured by ICP-MS, before and after application of Chelex. Additionally, the growth medium was supplemented with 50 *μ*M ZnCl_2_ and subsequently subjected to metal depletion. In both cases, Zn levels dropped below the detection limit after purification of the media by Chelex treatment.

Medium	Average Zn concentration [*μ*mol/L]
Neurobasal	1.42
Neurobasal + Chelex100	Below detection limit
Neurobasal + 50 *μ*M Zn	44.3
Neurobasal + 50 *μ*M Zn + Chelex100	Below detection limit

**Table 2:** Biometal profile seen in many ASD patients [3] derived from measurements of hair and serum samples.

Metal	Increase	Decrease
Cu	↑	
Fe		↓
Hg	↑	
Mn		↓
Pb	↑	
Se		↓
Zn		↓

## References

[1] Delorme R., Ey E., Toro R., Leboyer M., Gillberg C., Bourgeron T. (2013). Progress toward treatments for synaptic defects in autism. *Nature Medicine*.

[2] Grabrucker A. M. (2013). Environmental factors in autism. *Frontiers in Psychiatry*.

[3] Pfaender S., Grabrucker A. M. (2014). Characterization of biometal profiles in neurological disorders. *Metallomics*.

[4] Yasuda H., Tsutsui T. (2013). Assessment of infantile mineral imbalances in autism spectrum disorders (ASDs). *International Journal of Environmental Research and Public Health*.

[5] Lakshmi Priya M. D., Geetha A. (2011). Level of trace elements (copper, zinc, magnesium and selenium) and toxic elements (lead and mercury) in the hair and nail of children with autism. *Biological Trace Element Research*.

[6] Adams J. B., Audhya T., McDonough-Means S. (2013). Toxicological status of children with autism vs. neurotypical children and the association with autism severity. *Biological Trace Element Research*.

[7] Alexandrov P. N., Zhao Y., Pogue A. I. (2005). Synergistic effects of iron and aluminum on stress-related gene expression in primary human neural cells. *Journal of Alzheimer's Disease*.

[8] Bahbouth E., Siwek B., de Pauw-Gillet M. C., Sabbioni E., Bassleer R. (1993). Effects of trace metals on mouse B16 melanoma cells in culture. *Biological Trace Element Research*.

[9] Hernández R. B., Farina M., Espósito B. P., Souza-Pinto N. C., Barbosa F., Suñol C. (2011). Mechanisms of manganese-induced neurotoxicity in primary neuronal cultures: the role of manganese speciation and cell type. *Toxicological Sciences*.

[10] Rowles T. K., Womac C., Bratton G. R., Tiffany-Castiglioni E. (1989). Interaction of lead and zinc in cultured astroglia. *Metabolic Brain Disease*.

[11] van Landingham J. W., Fitch C. A., Levenson C. W. (2002). Zinc inhibits the nuclear translocation of the tumor suppressor protein p53 and protects cultured human neurons from copper-induced neurotoxicity. *NeuroMolecular Medicine*.

[12] White A. R., Barnham K. J., Huang X. (2004). Iron inhibits neurotoxicity induced by trace copper and biological reductants. *Journal of Biological Inorganic Chemistry*.

[13] Liebscher K., Smith H. (1968). Essential and nonessential trace elements. A method of determining whether an element is essential or nonessential in human tissue. *Archives of Environmental Health*.

[14] Grabrucker S., Jannetti L., Eckert M. (2014). Zinc deficiency dysregulates the synaptic ProSAP/Shank scaffold and might contribute to autism spectrum disorders. *Brain*.

[15] Grabrucker A. M., Vaida B., Bockmann J., Boeckers T. M. (2009). Synaptogenesis of hippocampal neurons in primary cell culture. *Cell and Tissue Research*.

[16] Sindreu C., Bayés À., Altafaj X., Pérez-Clausell J. (2014). Zinc transporter-1 concentrates at the postsynaptic density of hippocampal synapses. *Molecular Brain*.

[17] Bishop G. M., Dang T. N., Dringen R., Robinson S. R. (2011). Accumulation of non-transferrin-bound iron by neurons, astrocytes, and microglia. *Neurotoxicity Research*.

[18] Regan R. F., Jasper E., Guo Y., Panter S. S. (1998). The effect of magnesium on oxidative neuronal injury in vitro. *Journal of Neurochemistry*.

[19] White A. R., Multhaup G., Maher F. (1999). The Alzheimer's disease amyloid precursor protein modulates copper-induced toxicity and oxidative stress in primary neuronal cultures. *Journal of Neuroscience*.

[20] Savaskan N. E., Bräuer A. U., Kühbacher M. (2003). Selenium deficiency increases susceptibility to glutamate-induced excitotoxicity. *The FASEB Journal*.

[21] Aoshima K., Kasuya M. (1980). Interactions between mercuric chloride and sodium selenite on cultured rat cerebrum. *Toxicology Letters*.

[22] Bozym R. A., Chimienti F., Giblin L. J. (2010). Free zinc ions outside a narrow concentration range are toxic to a variety of cells in vitro. *Experimental Biology and Medicine*.

[23] Wang B., Du Y. (2013). Cadmium and its neurotoxic effects. *Oxidative Medicine and Cellular Longevity*.

[24] Ohtani-Kaneko R., Tazawa H., Yokosuka M., Yoshida M., Satoh M., Watanabe C. (2008). Suppressive effects of cadmium on neurons and affected proteins in cultured developing cortical cells. *Toxicology*.

[25] López E., Figueroa S., Oset-Gasque M. J., González M. P. (2003). Apoptosis and necrosis: two distinct events induced by cadmium in cortical neurons in culture. *British Journal of Pharmacology*.

[26] Gerspacher C., Scheuber U., Schiera G., Proia P., Gygax D., Di Liegro I. (2009). The effect of cadmium on brain cells in culture. *International Journal of Molecular Medicine*.

[27] Kowall N. W., Pendlebury W. W., Kessler J. B., Perl D. P., Beal M. F. (1989). Aluminum-induced neurofibrillary degeneration affecfts a subset of neurons in rabbit cerebral cortex, basal forebrain and upper brainstem. *Neuroscience*.

[28] Shaw C. A., Tomljenovic L. (2013). Aluminum in the central nervous system (CNS): toxicity in humans and animals, vaccine adjuvants, and autoimmunity. *Immunologic Research*.

[29] Kawahara M., Kato M., Kuroda Y. (2001). Effects of aluminum on the neurotoxicity of primary cultured neurons and on the aggregation of beta-amyloid protein. *Brain Research Bulletin*.

[30] Zhang L., Jin C., Liu Q. (2013). Effects of subchronic aluminum exposure on spatial memory, ultrastructure and L-LTP of hippocampus in rats. *Journal of Toxicological Sciences*.

[31] Jing Y., Wang Z., Song Y. (2004). Quantitative study of aluminum-induced changes in synaptic ultrastructure in rats. *Synapse*.

[32] Walton J. R. (2013). Aluminum's involvement in the progression of alzheimer's disease. *Journal of Alzheimer's Disease*.

[33] Harada M. (1995). Minamata disease: methylmercury poisoning in Japan caused by environmental pollution. *Critical Reviews in Toxicology*.

[34] Axelrad D. A., Bellinger D. C., Ryan L. M., Woodruff T. J. (2007). Dose-response relationship of prenatal mercury exposure and IQ: an integrative analysis of epidemiologic data. *Environmental Health Perspectives*.

[35] Dufault R., Schnoll R., Lukiw W. J. (2009). Mercury exposure, nutritional deficiencies and metabolic disruptions may affect learning in children. *Behavioral and Brain Functions*.

[36] Bose-O'Reilly S., McCarty K. M., Steckling N., Lettmeier B. (2010). Mercury exposure and children's health. *Current Problems in Pediatric and Adolescent Health Care*.

[37] Pieper I., Wehe C. A., Bornhorst J. (2014). Mechanisms of Hg species induced toxicity in cultured human astrocytes: Genotoxicity and DNA-damage response. *Metallomics*.

[38] Xu F., Farkas S., Kortbeek S. (2012). Mercury-induced toxicity of rat cortical neurons is mediated through N-methyl-D-Aspartate receptors. *Molecular Brain*.

[39] Landrigan P. J., Whitworth R. H., Baloh R. W. (1975). Neuropsychological dysfunction in children with chronic low level lead absorption. *Lancet*.

[40] Lanphear B. P., Hornung R., Khoury J. (2005). Low-level environmental lead exposure and children's intellectual function: an international pooled analysis. *Environmental Health Perspectives*.

[41] Audesirk T., Audesirk G., Ferguson C., Shugarts D. (1991). Effects of inorganic lead on the differentiation and growth of cultured hippocampal and neuroblastoma cells. *NeuroToxicology*.

[42] Hall A. C., Young B. W., Bremner I. (1979). Intestinal metallothionein and the mutual antagonism between copper and zinc in the rat. *Journal of Inorganic Biochemistry*.

[43] Francis G. L. (2010). Albumin and mammalian cell culture: implications for biotechnology applications. *Cytotechnology*.

[44] Frederickson C. J., Koh J. Y., Bush A. I. (2005). The neurobiology of zinc in health and disease. *Nature Reviews Neuroscience*.

[45] Huguet G., Ey E., Bourgeron T. (2013). The genetic landscapes of autism spectrum disorders. *Annual Review of Genomics and Human Genetics*.

[46] Guilmatre A., Huguet G., Delorme R., Bourgeron T. (2014). The emerging role of SHANK genes in neuropsychiatric disorders. *Developmental Neurobiology*.

[47] Grabrucker A. M., Schmeisser M. J., Schoen M., Boeckers T. M. (2011). Postsynaptic ProSAP/Shank scaffolds in the cross-hair of synaptopathies. *Trends in Cell Biology*.

[48] Jiang Y. H., Ehlers M. D. (2013). Modeling autism by SHANK gene mutations in mice. *Neuron*.

[49] Yoo J., Bakes J., Bradley C., Collingridge G. L., Kaang B.-K. (2014). Shank mutant mice as an animal model of autism. *Philosophical Transactions of the Royal Society B: Biological Sciences*.

[50] Gundelfinger E. D., Boeckers T. M., Baron M. K., Bowie J. U. (2006). A role for zinc in postsynaptic density asSAMbly and plasticity?. *Trends in Biochemical Sciences*.

[51] Baecker T., Mangus K., Pfaender S., Chhabra R., Boeckers T. M., Grabrucker A. M. (2014). Loss of COMMD1 and copper overload disrupt zinc homeostasis and influence an autism-associated pathway at glutamatergic synapses. *BioMetals*.

[52] Xie X., Smart T. G. (1994). Modulation of long-term potentiation in rat hippocampal pyramidal neurons by zinc. *Pflugers Archiv*.

[53] Lu Y.-M., Taverna F. A., Tu R., Ackerley C. A., Wang Y.-T., Roder J. (2000). Endogenous Zn^2+^ is required for the induction of long-term potentiation at rat hippocampal mossy fiber-CA3 synapses. *Synapse*.

[54] Grabrucker A. M., Knight M. J., Proepper C. (2011). Concerted action of zinc and ProSAP/Shank in synaptogenesis and synapse maturation. *The EMBO Journal*.

[55] Jan H. H., Chen I. T., Tsai Y. Y., Chang Y. C. (2002). Structural role of zinc ions bound to postsynaptic densities. *Journal of Neurochemistry*.

[56] Duffney L. J., Wei J., Cheng J. (2013). Shank3 deficiency induces NMDA receptor hypofunction via an actin-dependent mechanism. *The Journal of Neuroscience*.

[57] Won H., Lee H.-R., Gee H. Y. (2012). Autistic-like social behaviour in Shank2-mutant mice improved by restoring NMDA receptor function. *Nature*.

[58] Bai G., Hoffman P. W., van Dongen A. M. (2009). Transcriptional regulation of NMDA receptor expression. *Biology of the NMDA Receptor*.

[59] Jackson K. A., Valentine R. A., Coneyworth L. J., Mathers J. C., Ford D. (2008). Mechanisms of mammalian zinc-regulated gene expression. *Biochemical Society Transactions*.

[60] Hogstrand C., Zheng D., Feeney G., Cunningham P., Kille P. (2008). Zinc-controlled gene expression by metal-regulatory transcription factor 1 (MTF1) in a model vertebrate, the zebrafish. *Biochemical Society Transactions*.

[61] Langmade S. J., Ravindra R., Daniels P. J., Andrews G. K. (2000). The transcription factor MTF-1 mediates metal regulation of the mouse ZnT1 gene. *The Journal of Biological Chemistry*.

[62] Huang P. C., Smith B., Bohdan P., Corrigan A. (1980). Effect of zinc on cadmium influx and toxicity in cultured CHO cells. *Biological Trace Element Research*.

[63] El-Sayed A., Salem S. M., El-Garhy A. (2013). Protective effect of zinc against cadmium toxicity on pregnant rats and their fetuses at morphological, physiological and molecular level. *African Journal of Biotechnology*.

