# Supplementary material for: Effects of Trace Metal Profiles Characteristic for Autism on Synapses in Cultured Neurons
Source: Neural Plast. 2015 Feb 23;2015:985083. doi: 10.1155/2015/985083 (PMC4352758; doi:10.1155/2015/985083)
Supplement: Supplementary file 1 — Figure S1: Exemplary images of toxicity profiles of trace elements in primary hippocampal neuronal cell culture, glia cell culture and calculation of LD50 values for each trace element. Furthermore, definition of dendritic branching analysis and evaluation of osmotic pressure effects on neuronal cell health. Figure S2: Comparison of original and reconstituted NB+++ Medium and exemplary images of immunofluorescence intensities of Shank proteins under Ctrl, ASD2 and ASD2+Zn conditions. Further, analysis of antagonistic or synergistic relation between zinc and toxic trace elements and examination of presence of presynaptic zinc. Figure S3: Pure zinc deficiency is not causative for effects observed under ASD2 conditions. [file 985083.f1.pdf]

Figure S1: A,B) Exemplary images for the evaluation of cell health and dendritic branching after treatment with trace metals. Neurons were stained with DAPI and MAP2 and the total number of nuclei as well as the fraction of apoptotic nuclei per optic field was measured. Additionally, dendritic branching was calculated based on the analysis shown in (B). Dendritic branches showing signs of fragmentation were not counted. C) Treatment with Mannitol did not significantly affected cell health even at high concentration (>500 μM), excluding osmotic stress as major factor for the observed alterations. D) Based on the correlation between cell health and the concentration of a certain trace metal applied, LD50 concentrations were calculated for each metal. An exemplary graph for Cd is shown. E) Analysis of trace metal toxicity for glial cells. Glial cells were much more resistant to alterations in trace metal concentrations. F) Exemplary images corresponding to the evaluation of synapse density presented in Fig. 1D. Synapses were labeled using Bassoon fluorescence and the number of Bassoon positive puncta (green) measured per 10 μm dendrite length. Merged images show additional staining of nuclei using DAPI (blue) and MAP2 (red).

Figure S2: A) Comparison between Neurobasal™ medium (with B27, L-Glut, and pen/strep = NB+++) that was depleted for trace metals and reconstituted for all depleted trace metals (Mg, Ca, Fe, Zn) according to the manufacturer's indicated metal concentrations with unmodified Neurobasal™ medium (NB+++). The amount of cell death was calculated assessing the number of neuronal apoptotic nuclei (identified by MAP2 and DAPI staining) per optic field (from 5 fields of view) normalized against the total number of neurons per optic field. No significant differences between unmodified

(Ctrl NB+++ / ASD1 NB+++) and reconstituted media (Ctrl / ASD1) were observed. B) The number of primary, secondary and tertiary dendrites was investigated from 10 cell per condition. Cells were stained with MAP2 antibody. As signs of cell death, neurons show a fragmentation ("pinching off") dendrites, starting with branches more distal from the soma. Dendrites showing signs of fragmentation were not counted. No significant differences between unmodified and reconstituted media were observed. C) Synapses were labeled using Bassoon and Homer1b/c fluorescence and the number of immunoreactive puncta were measured per 10 μm dendrite length on primary dendrites (3 dendrites per cell, 10 cells in total per group). No significant differences between unmodified and reconstituted media were observed. D) Exemplary images of hippocampal neurons DIV 14 under Control, ASD2 and ASD2 + Zn conditions. Cells were stained for Shank1, Shank2 and Shank3 and the fluorescence intensity of synaptic Shank puncta was measured in 10 cells. Merged images show additional DAPI staining of the nuclei. E,F) Evaluation of alterations in neuronal cell health and synapse density due to the supplementation of 50 μM $ZnCl_2$ simultaneously with either 5 μM $CdCl_2$, 120 μM $CuCl_2$, 5 μM $HgCl_2$, 5 μM $PbCl_2$, or 6 μM $SeCl_4$ exposure. The number of neuronal apoptotic nuclei (identified by MAP2 and DAPI staining) per optic field (from 10 fields of view and n = 3 cultures) was assessed and normalized against the total number of neurons per optic field. F) Changes in synapse density were examined by labeling synapses using Bassoon as marker and the number of Bassoon positive puncta was measured per 10 μm dendrite length on primary dendrites (3 dendrites per cell, 10 cells in total per condition, n= 3 cultures). G) Staining for the pre-synaptic marker protein Bassoon and Zinpyr1 that labels free and weakly protein bound Zn reveals that pre-

synapses in hippocampal cell culture in non-Zn supplemented Neurobasal$^{TM}$ medium are devoid of pre-synaptic vesicular Zn due to the absence of co-localization of Bassoon (full arrow) and Zinpyr1/$Zn^{2+}$ (open arrow). The weak Zn signal apposed to the Bassoon staining most likely results from Zn enriched at post-synapses.

Figure S3: Hippocampal neurons were grown from DIV 10 to DIV 14 in cell culture media containing different sets of trace metals: ASD2 cells were grown in trace metal depleted Neurobasal$^{TM}$ medium that was reconstituted only for Mg and Ca, with addition of the putative toxic metals (0.5 μM Cd, Cu, Hg, and 2 μM Pb). Zinc deficient (ZnD) cells were grown in trace metal depleted Neurobasal$^{TM}$ medium that was reconstituted for all trace metals except Zn. A) The amount of cell death was calculated assessing the number of neuronal apoptotic nuclei (identified by MAP2 and DAPI staining) per optic field (from 5 fields of view) normalized against the total number of neurons per optic field. Compared to controls as well as compared to ZnD conditions, a significant reduction in cell health occurs in cells deficient in Fe and Zn and subjected to toxic metals (ASD2). B) The number of primary, secondary and tertiary dendrites was investigated from 10 cell per condition. Cells were stained with MAP2 antibody. Dendrites showing signs of fragmentation were not counted. Corresponding to the increase in cell death, neurons growing under ASD2 conditions showed significantly increased signs of dendritic fragmentation compared to control neurons and neurons grown under ZnD conditions. C) Synapses were labeled using Bassoon and Homer1b/c fluorescence and the number of immunoreactive puncta were measured per 10 μm dendrite length on primary dendrites (3 dendrites per cell, 10 cells in total per group). The

significant reduction reported under ASD2 conditions was not seen in neurons grown under ZnD for Bassoon and Homer1b/c. D) Expression levels of NMDA receptor subunits (GluN1, GluN2a, GluN2b) and SHANK genes (Shank1, Shank2, Shank3) were measured by qRT-PCR. Virtual mRNA concentrations are shown averaged from three replicates and normalized against HMBS. The significant decrease of GluN1 and GluN2a mRNA expression levels under ASD2 conditions was seen to a significant lesser amount under ZnD conditions. The significant reduction in gene expression levels observed in Shank family members under ASD2 conditions, similarly was not visible in neurons exposed to ZnD medium. E) Immunocytochemistry of hippocampal neurons DIV 14 grown under ASD2 and ZnD conditions. The fluorescence intensity of Shank positive puncta measured using antibodies specific for Shank1, Shank2 and Shank3 was quantified from 10 cells per condition. A significant reduction of the Zn bidning Shank2 and Shank3 is seen under ZnD but not ASD2 conditions. F) Analysis of protein expression levels in synaptic (P2) fractions of hippocampal neurons grown under control, ASD2 and ZnD conditions. The experiments were performed in triplicates, exemplary Western Blot bands are shown in the right panel. Protein expression levels were normalized against Actin or β-III Tubulin. The results show that the loss of NMDAR (GluN2a) shown before in ASD2 conditions occurs similarly in ZnD conditions since no significant difference can be detected. The loss of Shank in contrast is less pronounced in ZnD conditions.

Figure S1

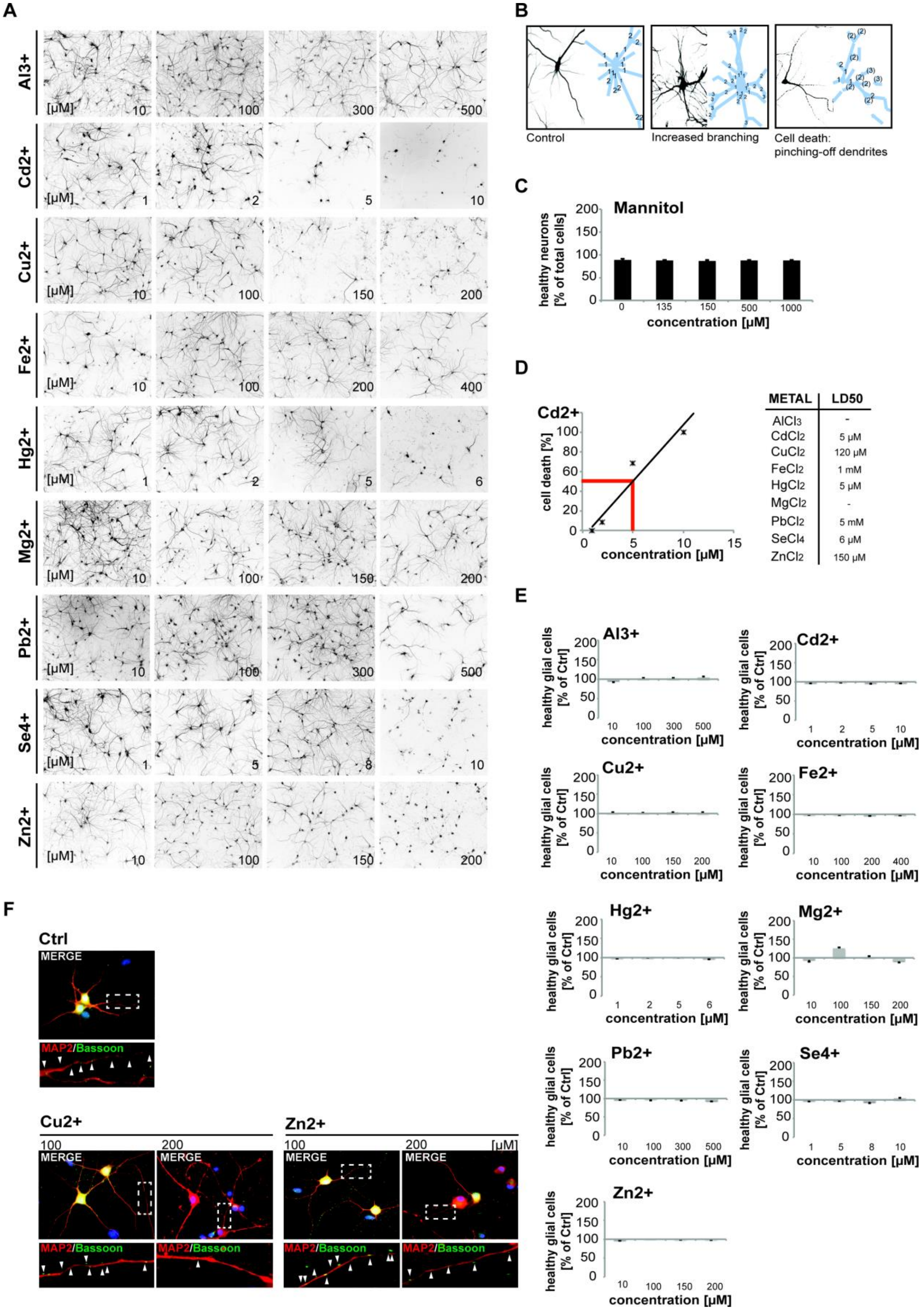

A
Al3+
[µM] 10 100 300 500
Cd2+
[µM] 1 2 5 10
Cu2+
[µM] 10 100 150 200
Fe2+
[µM] 10 100 200 400
Hg2+
[µM] 1 2 5 6
Mg2+
[µM] 10 100 150 200
Pb2+
[µM] 10 100 300 500
Se4+
[µM] 1 5 8 10
Zn2+
[µM] 10 100 150 200
B
Control
Increased branching
Cell death: pinching-off dendrites
C
Mannitol
healthy neurons [% of total cells]
0 135 150 500 1000
concentration [µM]
D
Cd2+
cell death [%]
concentration [µM]
METAL | LD50
AlCl3 -
CdCl2 5 µM
CuCl2 120 µM
FeCl2 1 mM
HgCl2 5 µM
MgCl2 -
PbCl2 5 mM
SeCl4 6 µM
ZnCl2 150 µM
E
healthy glial cells [% of Ctrl]
concentration [µM]
Al3+ Cd2+ Cu2+ Fe2+ Hg2+ Mg2+ Pb2+ Se4+ Zn2+
F
Ctrl
MERGE
MAP2/Bassoon
Cu2+
Zn2+
100 200 [µM]

Figure S2

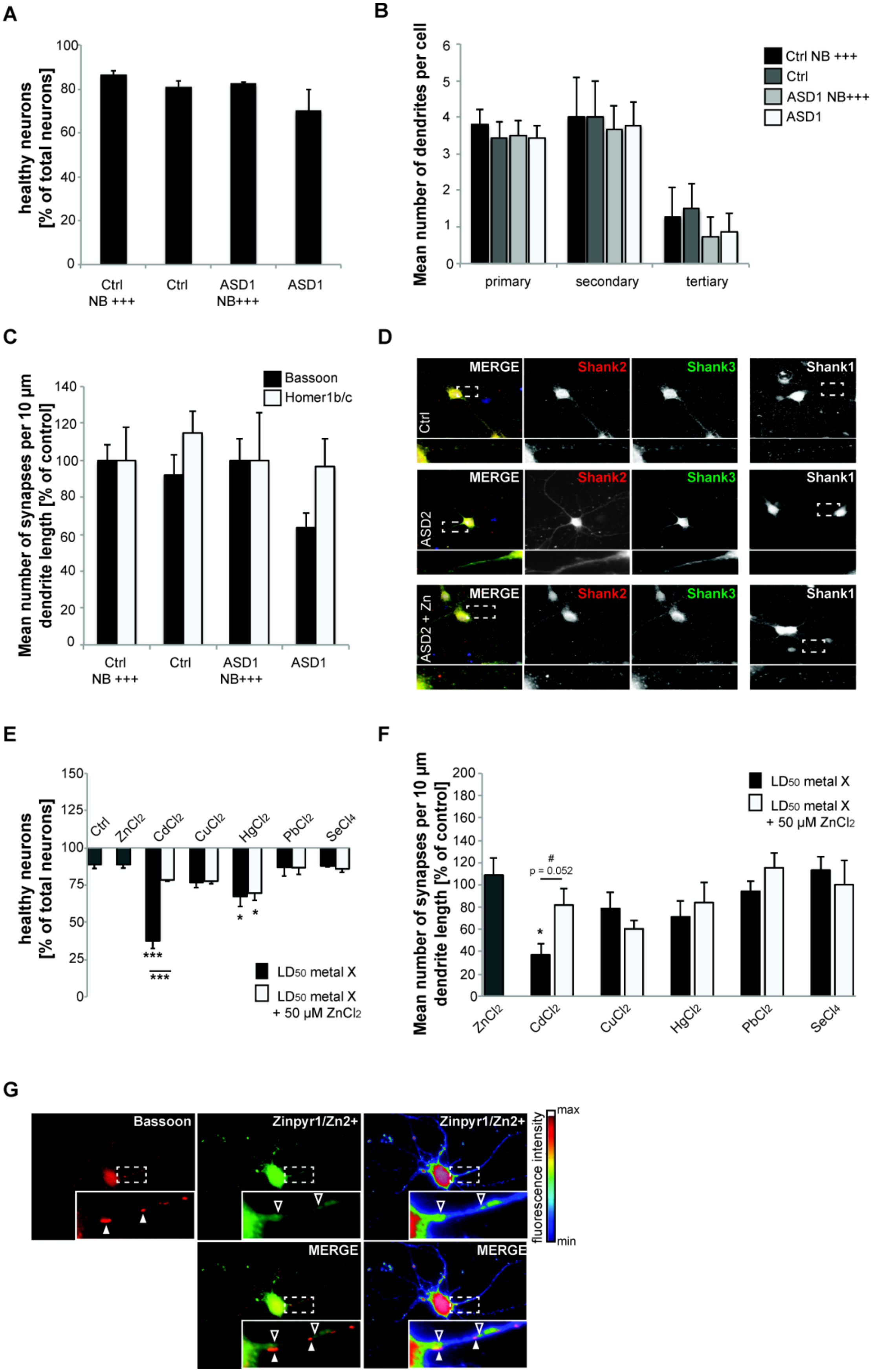
A
B
C
D
E
F
G
healthy neurons [% of total neurons]
Ctrl NB +++
Ctrl
ASD1 NB+++
ASD1
Mean number of dendrites per cell
primary
secondary
tertiary
Mean number of synapses per 10 µm dendrite length [% of control]
Bassoon
Homer1b/c
MERGE
Shank2
Shank3
Shank1
ASD2
ASD2 + Zn
LD50 metal X
LD50 metal X + 50 µM ZnCl2
p = 0.052
Zinpyr1/Zn2+
fluorescence intensity
max
min

Figure S3

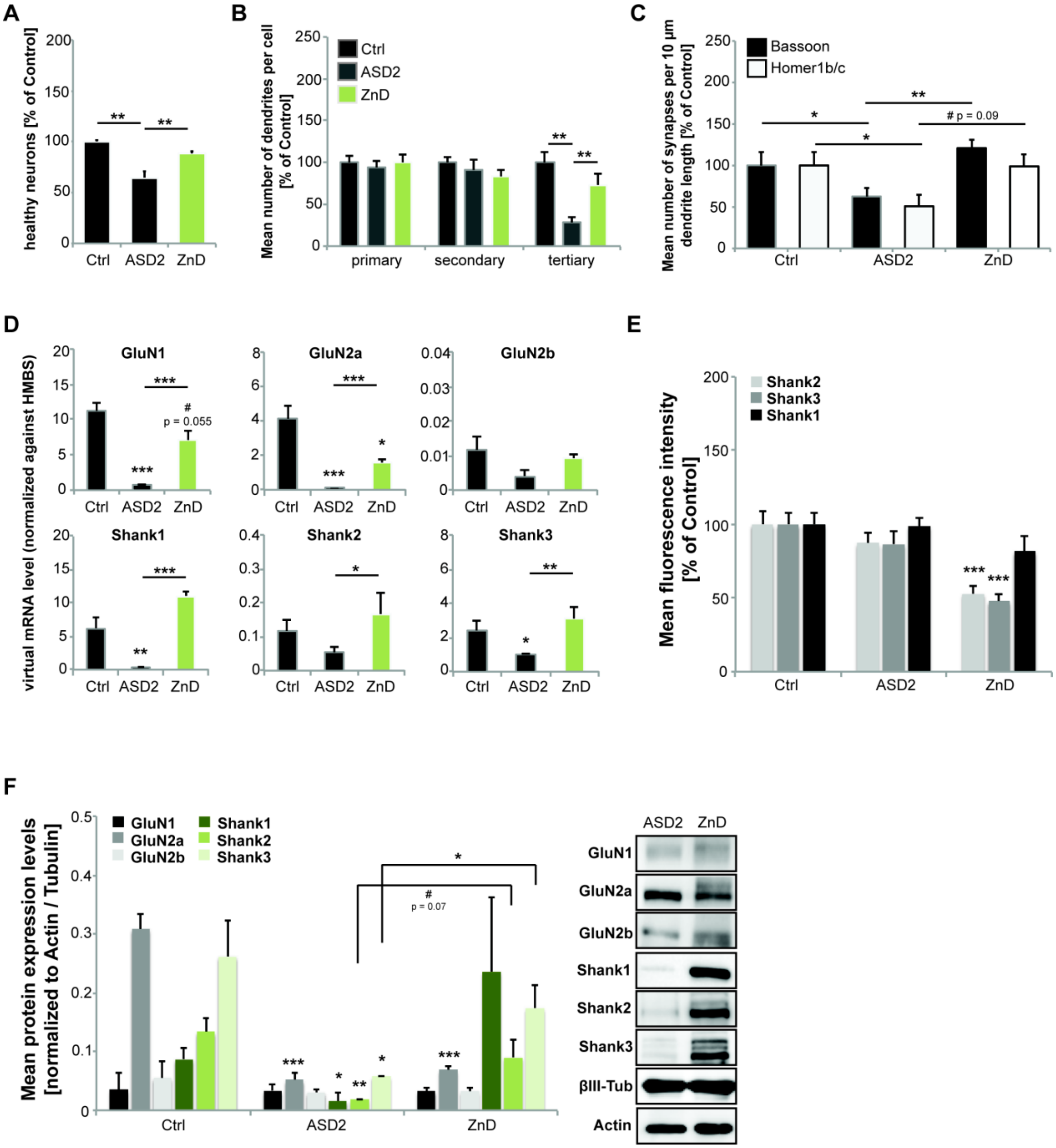

A
healthy neurons [% of Control]
Ctrl
ASD2
ZnD
B
Mean number of dendrites per cell [% of Control]
primary
secondary
tertiary
C
Bassoon
Homer1b/c
Mean number of synapses per 10 µm dendrite length [% of Control]
# p = 0.09
D
virtual mRNA level (normalized against HMBS)
GluN1
GluN2a
GluN2b
Shank1
Shank2
Shank3
p = 0.055
E
Mean fluorescence intensity [% of Control]
F
Mean protein expression levels [normalized to Actin / Tubulin]
p = 0.07
βIII-Tub
Actin
